# In Vitro and In Vivo Evaluations of β-Lactam/β-Lactamase Mono- and Combined Therapies against Carbapenem-Nonsusceptible Enterobacteriaceae in Taiwan

**DOI:** 10.3390/microorganisms8121981

**Published:** 2020-12-12

**Authors:** Tsung-Ying Yang, Ya-Ju Hsieh, Li-Ting Kao, Guan-Hong Liu, Shao-Hsuan Lian, Liang-Chun Wang, I-Ling Lin, Yu-Tzu Lin, Sheng-Fan Wang, Sung-Pin Tseng, Po-Liang Lu

**Affiliations:** 1 Department of Medical Laboratory Science and Biotechnology, College of Health Sciences, Kaohsiung Medical University, Kaohsiung 807378, Taiwan; zegma040899@gmail.com (T.-Y.Y.); jackjack90155@gmail.com (G.-H.L.); ladyrainicorn3838@gmail.com (S.-H.L.); linili@kmu.edu.tw (I.-L.L.); wasf1234@kmu.edu.tw (S.-F.W.); 2 Department of Medical Imaging and Radiological Sciences, Kaohsiung Medical University, Kaohsiung 807378, Taiwan; yjhsieh@kmu.edu.tw; 3 Orthopedic Research Center, College of Medicine, Kaohsiung Medical University, Kaohsiung 807378, Taiwan; gaufang@gmail.com; 4 Department of Marine Biotechnology and Resources, National Sun Yat-sen University, Kaohsiung 80424, Taiwan; marknjoy@g-mail.nsysu.edu.tw; 5 Department of Laboratory Medicine, Kaohsiung Medical University Hospital, Kaohsiung 807377, Taiwan; 6 Department of Medical Laboratory Science and Biotechnology, China Medical University, Taichung 40402, Taiwan; yutzulin@mail.cmu.edu.tw; 7 Center for Tropical Medicine and Infectious Disease Research, Kaohsiung Medical University, Kaohsiung 807378, Taiwan; 8 School of Post-Baccalaureate Medicine, College of Medicine, Kaohsiung Medical University, Kaohsiung 807378, Taiwan; 9 Division of Infectious Diseases, Department of Internal Medicine, Kaohsiung Medical University Hospital, Kaohsiung 807377, Taiwan; 10 Center for Liquid Biopsy and Cohort Research, Kaohsiung Medical University, Kaohsiung 807378, Taiwan

**Keywords:** CR Enterobacteriaceae, combination therapy, molecular epidemiology of antimicrobial resistance

## Abstract

Increasing carbapenem resistance rates worldwide underscored the urgent need of novel antimicrobials. Ceftazidime–avibactam and aztreonam–avibactam combinations are developed to combat carbapenem resistance, but biological and geographic variations must be considered for antibiotic susceptibility patterns varied. Thus, we sought to assess the susceptibilities of ceftazidime–avibactam and aztreonam–avibactam against 660 carbapenem-nonsusceptible Enterobacteriaceae isolates (472 *Klebsiella pneumoniae* and 188 *Escherichia coli*) collected during an earlier Taiwan surveillance study. Agar dilution method was used to determine ceftazidime–avibactam and aztreonam–avibactam susceptibility. Metallo-carbapenemase’s contribution to resistance were investigated with EDTA addition. The in vivo efficacies were evaluated using a *Caenorhabditis elegans* model. High susceptibility rates were observed for ceftazidime–avibactam and aztreonam–avibactam against the 472 carbapenem-nonsusceptible *K. pneumoniae* (CnsKP) (85.2% and 95.3%, respectively) and 188 carbapenem-nonsusceptible *E. coli* (CnsEC) isolates (91.5% and 94.1%, respectively). For non-metallo-carbapenemase producers, the susceptibility rates for ceftazidime–avibactam were 93.6% for CnsKP and 97.7% for CnsEC, whereas only 7.1% CnsKP and 11.1% CnsEC in metallo-carbapenemase producers were susceptible to ceftazidime–avibactam. Of all isolates, 95.3% CnsKP and 94.1% CnsEC were susceptible to aztreonam–avibactam. In *C. elegans* model, ceftazidime–avibactam and aztreonam–avibactam revealed effective against a *bla*_KPC_-producing *K. pneumoniae* isolate in vivo. Our results propose a positive therapeutic approach for both combinations against carbapenem-nonsusceptible Enterobacteriaceae in Taiwan.

## 1. Introduction

Due to the rapid dissemination of resistant genes and the over-prescription and overconsumption of carbapenems, health care professionals all over the world are facing challenges associated with carbapenem-resistant Enterobacteriaceae (CRE) infections, with treatments costing billions of dollars [1]. Carbapenem resistance mechanisms are associated with the production of transmittable carbapenemases, the loss of porins in combination with *bla*_AmpC_ β-lactamase overexpression, and active efflux pumps [2]. Global epidemiological studies in the Asia–Pacific region, the Indian subcontinent, Europe, North America, and Latin America indicate carbapenem resistance rates of up to 58.6% in Enterobacteriaceae, with significantly higher rates in Europe and India [3]. In Taiwan, 10.5% (71/673) *K. pneumoniae* bloodstream isolates collected in 2017 were not susceptible to at least one carbapenem [4].

Ceftazidime-avibactam, a β-lactam-plus-β-lactamase inhibitor combination that received US FDA approval in 2015, has been described as having anti-CRE efficacy, except for metallo-beta-lactamase producers [5]. Another CRE infection treatment option that is currently in phase III clinical trials is the combination of aztreonam and avibactam (NCT03580044 and NCT03329092) [6]. However, local antibiotic susceptibility patterns are important when prescribing these new agents empirically and before the metallo-beta-lactamase producers were identified [7]. In a SIDERO-WT-2014 study, different resistance rates in North America (3.3%) and Europe (28.1%) were observed for ceftazidime–avibactam in meropenem-nonsusceptible Enterobacteriaceae [7]. In another SIDERO-WT-2014 study, the authors reported that the KPC-type enzymes were the dominant carbapenemase carriage in both North America and Europe, but metallo-carbapenemases (NDM, VIM, or IMP) were mainly found in European isolates [8]. From their results of antimicrobial susceptibility testing, ceftazidime-avibactam was noted with poor activities against metallo-carbapenemase producers. For the present research we assessed the in vitro and in vivo efficacies of ceftazidime-avibactam and aztreonam-avibactam against 660 carbapenem-nonsusceptible Enterobacteriaceae isolates collected as part of a nationwide surveillance project in Taiwan. Bioinformatic analyses were performed to clarify our results and to identify factors affecting susceptibility.

## 2. Materials and Methods

### 2.1. Bacterial Isolate Collection

The collection of 660 carbapenem-nonsusceptible Enterobacteriaceae isolates, including 472 nonduplicated carbapenem-nonsusceptible *K. pneumoniae* (CnsKP) isolates in 2014 (472/660, 71.5%) and 188 nonduplicated carbapenem-nonsusceptible *E. coli* (CnsEC) isolates in 2012–2015 (188/660, 18.5%), was completed as part of a national surveillance study involving 16 Taiwanese hospitals [9,10]. The primary isolation source was urine (*n* = 251, 38.0%), followed by sputum/endotracheal aspirates (*n* = 129, 19.6%), blood (*n* = 56, 8.5%), wounds/pus (*n* = 61, 9.2%), stool/rectal swabs (*n* = 35, 5.3%), bile (*n* = 33, 5.0%), ascites (*n* = 26, 3.9%), and abscesses (*n* = 13, 2.0%). Sources for the other 56 isolates (8.5%) included percutaneous transhepatic cholangiography and drainage (PCTD), central venous pressure (CVP) tips, gas sampling lines, and milk. Carbapenem nonsusceptibility was defined as intermediate resistance or resistance to at least one carbapenem in accordance with Clinical and Laboratory Standards Institute (CLSI) guidelines [11].

### 2.2. Antimicrobial Susceptibility Testing

Broth microdilution (Sensititre, Trek Diagnostic Systems, Cleveland, OH, USA) was used to determine the susceptibilities of 18 antimicrobial agents: ampicillin, cefazolin, cefoxitin, cefotaxime, ceftazidime, ceftriaxone, cefepime, imipenem, doripenem, meropenem, ertapenem, aztreonam, piperacillin–tazobactam, levofloxacin, ciprofloxacin, amikacin, gentamicin, and trimethoprim/sulfamethoxazole. Results are reported according to CLSI-established minimum inhibitory concentration (MIC) breakpoints [11].

Standard agar dilution tests were used to measure the MICs of β-lactam/β-lactamase inhibitor combinations. Avibactam (AVI) was assessed at a concentration of 4 mg/L in combination with 2-fold dilutions of ceftazidime (CAZ) or aztreonam (AZT) [11]. CAZ and AZT monotherapy MIC values were also determined using the agar dilution method. In all, 23 isolates carrying various metallo-carbapenemases were used to estimate MIC values with or without EDTA at 320 mg/L [12].

### 2.3. β-. Lactamase and Carbapenemase Gene Detection

PCR was performed to determine the presence of extended-spectrum β-lactamase (ESBL) genes (*bla*_CTX-M-G1_, *bla*_CTX-M-G2_, and *bla*_CTX-M-G9_), carbapenemase genes (*bla*_KPC_, *bla*_NDM_, *bla*_IMP_, *bla*_NMC_, *bla*_SME_, *bla*_VIM_, *bla*_SPM-1_, *bla*_GIM-1_, *bla*_SIM-1_, *bla*_IMI_, *bla*_GES_, and *bla*_OXA-48_), and plasmid-mediated *bla*_AmpC_ genes (*bla*_DHA_ and *bla*_CMY_) [13,14]. All detection activity involved respective gene controls.

### 2.4. In Vivo Caenorhabditis elegans Study

*C. elegans* strain N2 was used to evaluate the treatment effects of CAZ-AVI and AZT-AVI combinations against the KPC-producing *K. pneumoniae* clinical isolate CRE-1462, a member of sequence type 11, the most prevalent in Taiwan [10]. Nematodes were maintained at 20 °C on growth medium agar plates with the OP50 non-toxic *E. coli* laboratory strain. Protocols are described in detail in an earlier report [15]. Briefly, 700–1000 growth-synchronized L4 worms were infected with CRE-1462 for 3 days, and 40 infected worms were transferred onto nematode growth medium (NGM) agar with either a placebo, β-lactam alone (CAZ or AZT), or a β-lactam/β-lactamase inhibitor combination (CAZ-AVI or AZT-AVI). Antibiotic concentrations were 8 mg/L for CAZ and 4 mg/L for AZT, alone or in combination. Avibactam was examined at a fixed concentration of 4 mg/L in combination with individual antibiotics. Nematode survival was monitored daily; surviving worms were transferred onto new plates and treated at the same concentrations. Assays were performed in triplicate.

### 2.5. Statistical Analyses

Antimicrobial susceptibility test and gene detection results were visualized as ggplot2 package heatmaps using RStudio (version 1.1.453). The log_2_-transformed MIC values were used for statistical analyses using GraphPad Prism Version 7.0 software (San Diego, CA, USA) with paired *t*-tests. Log-rank (Mantel–Cox) tests in the same software package were used to create Kaplan–Meier survival test curves.

## 3. Results

### 3.1. Enterobacteriaceae Isolates

**Figure 1:**
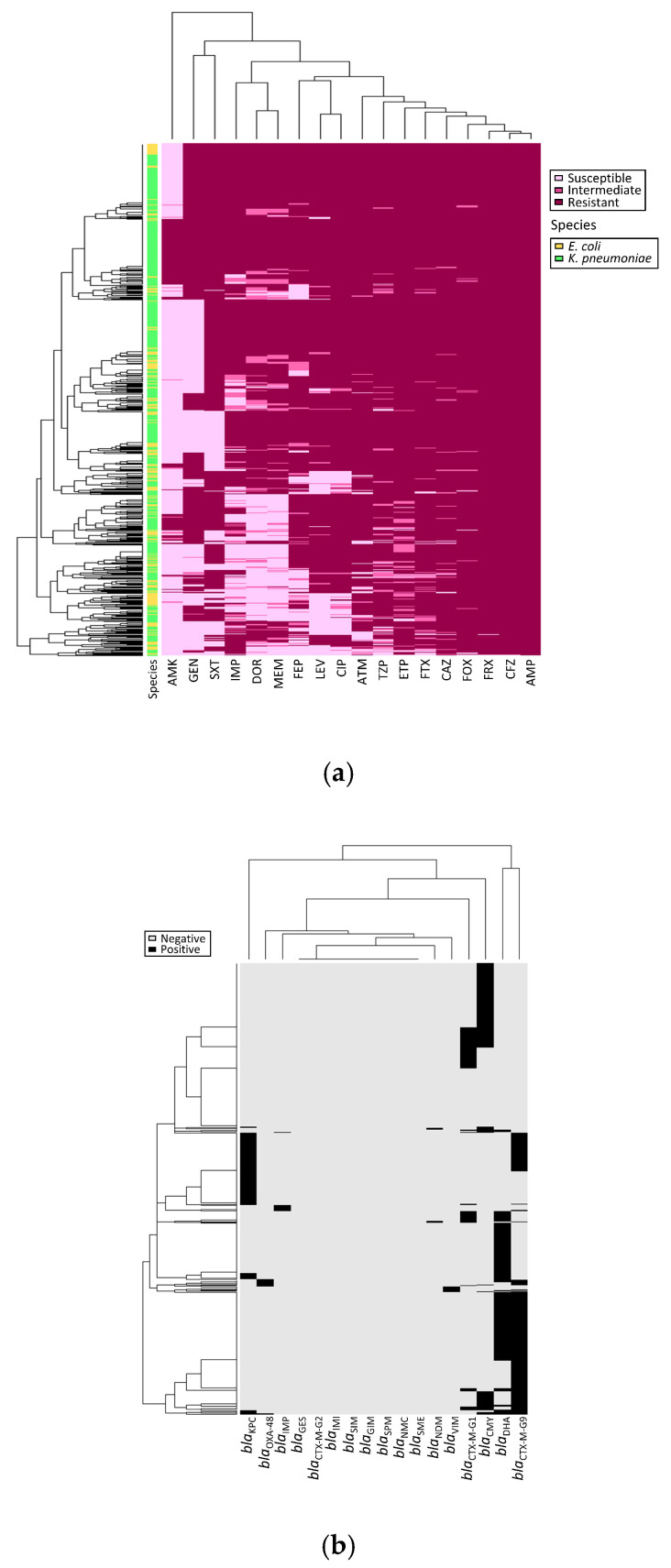
**(a) Antimicrobial susceptibility profile and (b) gene detection heatmap for 660 carbapenem-nonsusceptible Enterobacteriaceae isolates.** Abbreviations: AMK, amikacin; GEN, gentamicin; SXT, trimethoprim/sulfamethoxazole; IMP, imipenem; DOR, doripenem; MEM, meropenem; FEP, cefepime; LEV, levofloxacin; CIP, ciprofloxacin; ATM, aztreonam; TZP, piperacillin–tazobactam; ETP, ertapenem; FTX, cefotaxime; CAZ, ceftazidime; FOX, cefoxitin; FRX, ceftriaxone; CFZ, cefazolin; AMP, ampicillin. Indicated are negative and positive PCR detection results for each gene.

Our antimicrobial susceptibility test results revealed high antibiotic-resistance rates in 660 carbapenem-nonsusceptible Enterobacteriaceae isolates (472 CnsKP and 188 CnsEC). The list of antibiotics and their resistance rates includes ampicillin (660/660, 100%), cefazolin (659/660, 99.8%), ceftriaxone (658/660, 99.7%), ceftazidime (654/660, 99.1%), ertapenem (648/660, 98.2%), cefotaxime (646/660, 97.9%), cefoxitin (645/660, 97.7%), piperacillin–tazobactam (632/660, 95.8%), aztreonam (619/660, 93.8%), ciprofloxacin (586/660, 88.8%), levofloxacin (556/660, 84.2%), cefepime (547/660, 82.9%), meropenem (517/660, 78.3%), imipenem (516/660, 78.2%), trimethoprim/sulfamethoxazole (511/660, 77.4%), and doripenem (493/660, 74.7%) (Figure 1a). A moderate level of resistance was found in gentamicin (379/660, 57%). Amikacin exhibited surprisingly strong antibacterial activity (139/660, 21.1% resistance).

Detection results for *bla*_ESBL_ and *bla*_AmpC_ indicate that 92 isolates carried *bla*_CTX-M-G1_ (92/660, 13.9%), 250 carried *bla*_CTX-M-G9_ (250/660, 37.9%), 165 *bla*_CMY_ (165/660, 25.0%), and 222 *bla*_DHA_ (222/660, 33.6%) (Figure 1b). The most common carbapenemase gene was *bla*_KPC_, (123/660, 18.6%), followed by *bla*_OXA-48_ (13/660, 2.0%), *bla*_IMP_ (10/660, 1.5%), *bla*_VIM_ (8/660, 1.2%), and *bla*_NDM_ (5/660, 0.8%).

### 3.2. In Vitro β-lactam with β-lactamase Inhibitor Activity

According to our in vitro results, ceftazidime with avibactam and aztreonam with avibactam were significantly more powerful than their respective monotherapies (Table 1). Significant in vitro effects of ceftazidime–avibactam and aztreonam–avibactam were also noted in class A and D carbapenemase-producing *K. pneumoniae* isolates, but not in class B. The mean log_2_ MIC differences and their 95% confidence intervals (95% CIs) of ceftazidime–avibactam against *K. pneumoniae* isolates with Class A and Class D carbapenemases were −5.2 (−5.4, −5.0; *p* < 0.0001) and −4.8 (−5.7, −3.9; *p* < 0.0001), respectively; those of aztreonam–avibactam against *K. pneumoniae* isolates with Class A and Class D carbapenemases were revealed as −6.3 (−6.5, −6.2; *p* < 0.0001) and −6.0 (−7.2, −4.8; *p* < 0.0001). Among 14 class B carbapenemase-producing *K. pneumoniae* isolates, no statistically significant differences were noted between the MIC values for ceftazidime alone and ceftazidime combined with avibactam. In contrast, a significant increase (*p* < 0.0001) in effectiveness was noted for aztreonam combined with avibactam, with susceptibility of 92.9% (13/14), reductions in both MIC_50_ (from >32 to 0.125 mg/L) and MIC_90_ values (from >32 to 0.5 mg/L), and a decrease in the log_2_ MIC value (−6.0; 95% CI, −8.2, −3.8; *p* < 0.0001). Among 188 isolates of carbapenem-resistant *E. coli*, similarly significant in vitro effects of ceftazidime–avibactam and aztreonam–avibactam were noticed. The mean log_2_ MIC difference and their 95% confidence intervals (95% CIs) of ceftazidime–avibactam against *E. coli* isolates with Class A carbapenemases were −5.3 (−8.2, −2.5; *p* < 0.0001); those of aztreonam–avibactam against *E. coli* isolates with Class A carbapenemases were revealed as −6.3 (−6.5, −6.2; *p* < 0.0001). Among 9 class B carbapenemase-producing *E. coli* isolates, no difference in MICs were noted for ceftazidime combined with avibactam (*p* = 0.2953) compared to ceftazidime monotherapy. In contrast, a significant decreased MIC (*p* < 0.0001) and increased susceptibility (11.1% to 100%) was observed in vitro for aztreonam combined with avibactam compared to aztreonam alone, with a significant difference in log_2_ MIC (−8.1; 95% CI, −9.6, −6.6; *p* < 0.0001).

**Figure 2:**
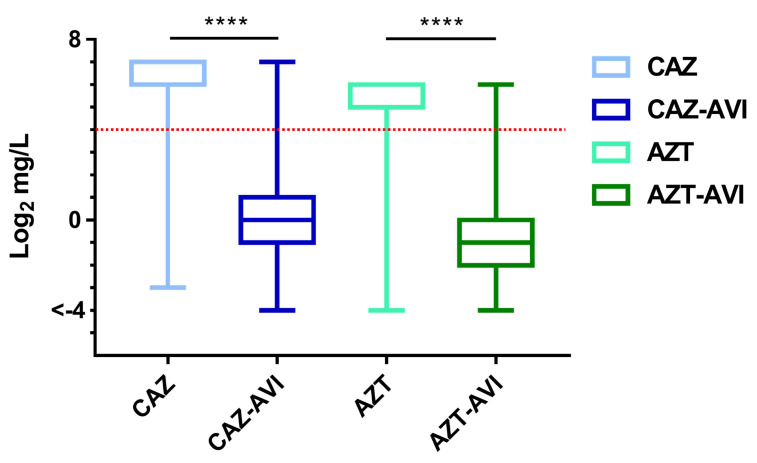
**MIC distribution box plots for the four regimens tested in this study.** Red dotted line indicates resistance breakpoints for each agent according to CLSI guidelines. From top to bottom, horizontal lines indicate maximum, third quartile (Q3, 75%), medium (50%), first quartile (Q1, 25%), and minimum MIC values. Abbreviations: CAZ, ceftazidime; CAZ-AVI, ceftazidime with avibactam; AZT, aztreonam; AZT-AVI, aztreonam with avibactam. ****, *p* < 0.0001.

Figure 2 shows box-plot MIC distribution data for four regimens: ceftazidime, ceftazidime with avibactam, aztreonam, and aztreonam with avibactam. Compared to their monotherapies, the combined therapies resulted in significant improvements in antibacterial activity in 660 carbapenem-nonsusceptible Enterobacteriaceae clinical isolates (*p* > 0.0001 for both). For ceftazidime alone, a large proportion of isolates possessed MIC values above the CLSI resistance breakpoint (Figure 2, red dotted line), while the combination of ceftazidime with avibactam triggered a statistically significant decrease (*p* < 0.0001) in MIC distribution, with more than 75% of all isolates showing MIC values below the breakpoint. A similar result was found for aztreonam with avibactam (*p* < 0.0001).

**Figure 3:**
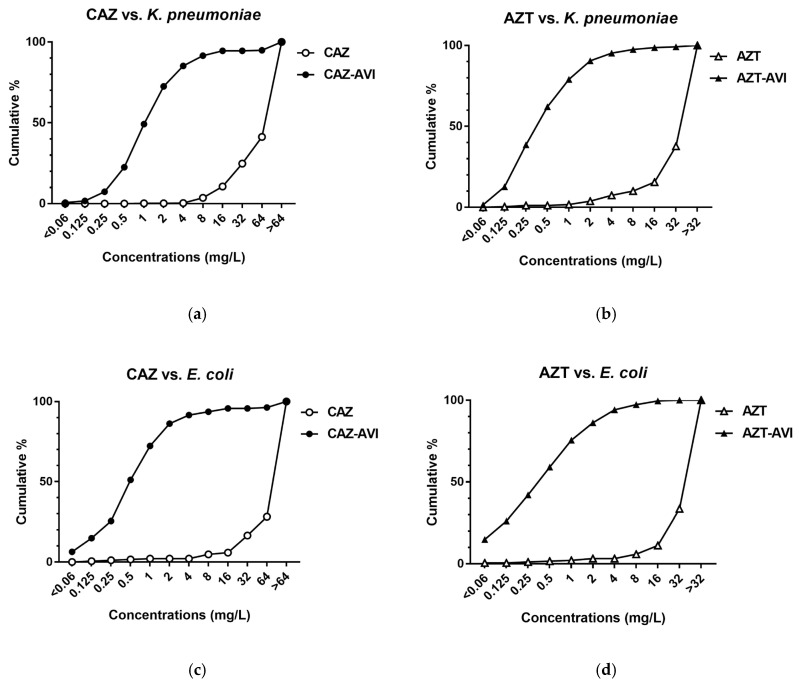
**Cumulative MIC susceptibility curves against 472 CnsKP (a,b) and 188 CnsEC isolates (c,d).** Data shown are for ceftazidime (**a**,**c**) and aztreonam (**b**,**d**). Abbreviations: CAZ, ceftazidime; CAZ-AVI, ceftazidime with avibactam; AZT, aztreonam; AZT-AVI, aztreonam with avibactam.

Cumulative MIC susceptibility curves are shown in Figure 3. Among the CnsKP isolates, ceftazidime and aztreonam susceptibility percentages were 0.4% (2/472) and 7.4% (35/472), respectively (Figure 3a,b). Leftward shifts were noted in 85.2% (402/472) of the same isolates following treatment with the ceftazidime–avibactam combination, and in 95.3% (450/472) following treatment with the aztreonam–avibactam combination (Figure 3a,b). Among the 188 CnsEC isolates, susceptibility values for ceftazidime and aztreonam monotherapies were 2.1% (4/188) and 3.2% (6/188), respectively (Figure 3c,d). Ceftazidime and aztreonam susceptibility values decreased to 91.5% (172/188) and 94.1% (177/188) when combined with avibactam, also respectively (Figure 3c,d). Combined, our data suggest that avibactam restored the antibacterial efficacies of ceftazidime and aztreonam.

### 3.3. Combination Therapy Efficacy Against Metallo-Carbapenemase Producers

**Figure 4:**
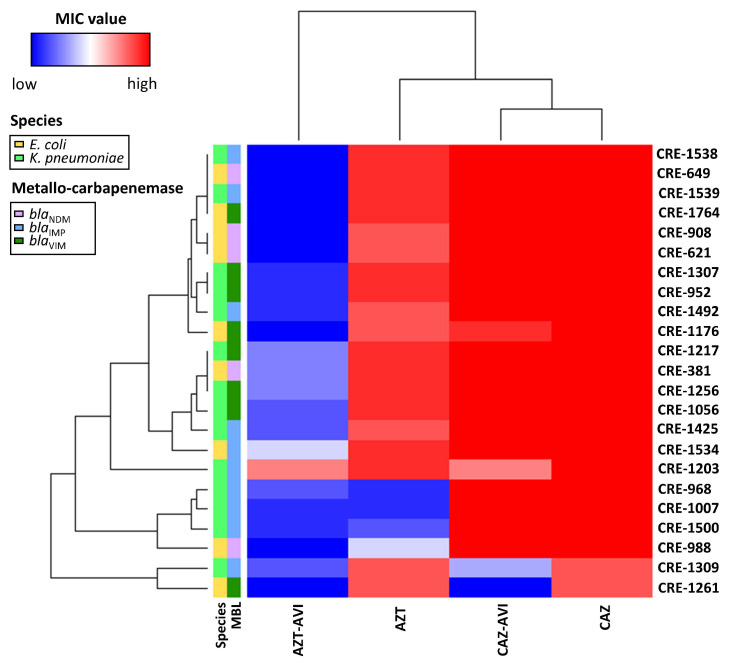
**Metallo-β-lactamase (MBL) heatmap showing MIC values for four regimens against 23 metallo-carbapenemase producers.** Colors indicate values from low (blue) to high (red). Abbreviations: CAZ, ceftazidime; CAZ-AVI, ceftazidime with avibactam; AZT, aztreonam; AZT-AVI, aztreonam with avibactam.

Low antibacterial activity for the combination of ceftazidime with avibactam was observed in 23 class B metallo-carbapenemase producers (Table 1). The heatmap shown as Figure 4 presents MIC values for all 23, along with their species and carbapenemase classifications. Among them, *bla*_IMP_ and *bla*_VIM_ were found in 9 and 5 CnsKP isolates, respectively, and *bla*_IMP_, *bla*_VIM_ and *bla*_NDM_ were found in 1, 3, and 5 CnsEC isolates, also respectively (Figure 4). Similar MIC patterns were observed for ceftazidime alone and ceftazidime with avibactam, indicating weak effectiveness against metallo-carbapenemase producers. In contrast, strong in vitro activity against the same isolates was observed for the combination of aztreonam with avibactam.

**Figure 5:**
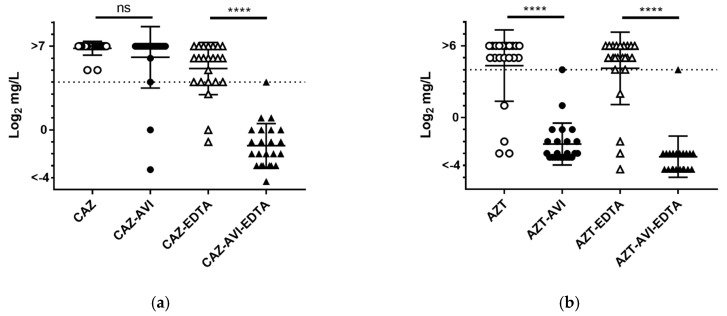
**MIC distributions of (a) ceftazidime (hollow circles) and (b) aztreonam (hollow circles) and their respective combination therapies (filled circles) following the addition of EDTA (triangles).** Abbreviations: CAZ, ceftazidime; CAZ-AVI, ceftazidime with avibactam; AZT, aztreonam; AZT-AVI, aztreonam with avibactam. ns, no statistical significance; ****, *p* < 0.0001.

EDTA was added to agar at various concentrations for each regimen to assess metallo-carbapenemase contributions to the efficacies of the three combination therapies. A statistically significant difference was observed between ceftazidime and ceftazidime with avibactam in the presence of EDTA (*p* < 0.0001), but not in its absence (Figure 5a). We found that EDTA inhibited metallo-carbapenemase and recovered the strength of ceftazidime with avibactam in vitro. The presence of *bla*_ESBL_ genes in the 23 metallo-carbapenemase producers might explain the reduction in activity observed in ceftazidime alone. Avibactam was capable of restoring the antibacterial efficacy of aztreonam in either the presence or absence of EDTA, with significant decreases in MIC values (both *p* < 0.0001) (Figure 5b). It did not have the same effect when added to ceftazidime (Figure 5a).

### 3.4. In Vivo C. Elegans Study

**Figure 6:**
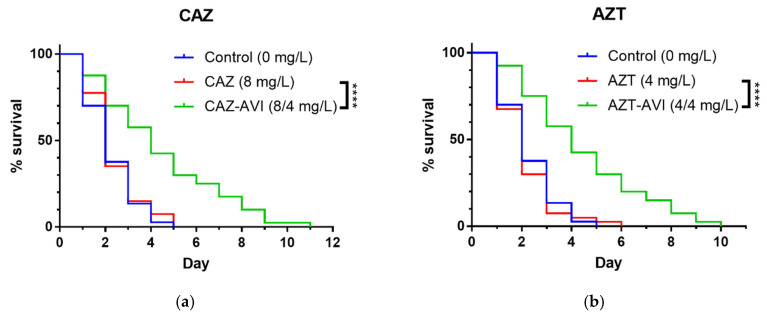
***C. elegans* survival curves. Infected nematodes (*n* = 40) were treated with (a) ceftazidime or ceftazidime with avibactam, (b) aztreonam or aztreonam with avibactam.** Nematodes were consistently treated with avibactam at a concentration of 4 mg/L. Abbreviations: CAZ, ceftazidime; CAZ-AVI, ceftazidime with avibactam; AZT, aztreonam; AZT-AVI, aztreonam with avibactam. ****, *p* < 0.0001.

A *C. elegans* model was used to evaluate the in vivo efficacies of the two combination therapies against a randomly selected carbapenem-resistant *K. pneumoniae* isolate (CRE-1462) carrying the *bla*_KPC_ gene, the most common carbapenemase gene in Taiwan. Compared to CRE-1462-infected nematodes subjected to ceftazidime monotherapy, the median survival time of nematodes treated with the ceftazidime–avibactam combination increased significantly (*p* < 0.0001) (Figure 6a). A strong treatment effect was also noted for the aztreonam with avibactam group (*p* < 0.0001), with a significant right-shift curve compared to the single-agent therapy group (Figure 6b).

Median survival time for infected nematodes either treated with ceftazidime monotherapy or untreated was two days (Table 2). Treatment with the ceftazidime–avibactam combination extended median survival to 4 days, with a significant 0.472 hazard ratio (HR) reduction (95% confidence interval (CI) 0.295 to 0.756) (*p* < 0.0001). Compared to the aztreonam monotherapy group, median time for the combined aztreonam–avibactam group increased from 2 to 4 days (HR 0.420; 95% CI 0.260 to 0.679) (*p* < 0.0001). In sum, our data indicate that both combination therapies were capable of rescuing the *C. elegans* model infected with a carbapenem-resistant *K. pneumoniae* isolate.

## 4. Discussion

There are currently many reports of carbapenem-resistant Enterobacteriaceae (CRE) worldwide, with limited clinical therapeutic options due to multidrug resistance [2,16,17,18]. In one international study of 267 metallo-carbapenemase Enterobacteriaceae isolates, resistance rates to ceftazidime, meropenem, piperacillin-tazobactam and levofloxacin ranged from 71.2% to 98.5%, compared to 10.9% for tigecycline and 12.2% for colistin [19]. In an earlier study conducted in Shanghai, 109 carbapenem-resistant *K. pneumoniae* isolates were found to be highly resistant (85.3–98.2%) to 13 of 18 tested antimicrobials; in that study, colistin expressed 96.3% susceptibility [20]. In another report from China, high resistance rates (92.7–100%) were observed for 11 of 17 antimicrobial agents tested with 41 carbapenem-resistant *K. pneumoniae* isolates [21]. An epidemiological investigation in Taiwan found that over 70% of CnsEC isolates were resistant to 9 antimicrobials, with less than 10% resistant to colistin, amikacin, or tigecycline [9]. In the present study we determined high resistance rates (77.4–100%) in 13 antimicrobials, with robust antibacterial activity only observed for amikacin (139/660 isolates, 19.1%). Despite the combined evidence for amikacin, colistin, or tigecycline as alternative therapies for CRE infections (Figure 1a), increasing resistance rates indicate an urgent need for novel antimicrobials.

Avibactam, a first-in-class serine β-lactamase inhibitor [22], is part of the ceftazidime–avibactam combination approved by the FDA in 2015 [5]. One research team reported MIC values ranging from 0.12 to >64 for ceftazidime–avibactam against 30 meropenem-nonsusceptible Enterobacteriaceae samples collected in North America, with a low resistance rate of 3.3% (1/30) [7]. In comparison, a moderate (28.1%) resistance rate was reported in a European study involving 139 isolates (MIC values from <0.06 to >64), and Kazmierczak et al., reported MIC values of 0.12 to >64 mg/L (including an MIC_50_ of 1 mg/L and MIC_90_ of >64 mg/L) for ceftazidime-avibactam against 151 meropenem-resistant Enterobacteriaceae isolates collected in North America and Europe (24.5% resistance rate; 37/151) [8]. In our study we noted significant improvement in the distribution of ceftazidime–avibactam MIC values against carbapenem-nonsusceptible Enterobacteriaceae compared to ceftazidime monotherapy (*p* < 0.0001) (Table 1, Figure 2), with high susceptibilities noted in both CnsEC (91.5%, 172/188) and CnsKP (85.2%, 402/472) (Figure 3a,c).

The aztreonam–avibactam combination is currently undergoing phase III clinical trials as an option for treating carbapenem-resistant Enterobacteriaceae infections [23]. In a study involving Gram-negative pathogens collected in 2012 and 2013 from 190 medical centers in 39 countries, 577 of 23,516 Enterobacteriaceae isolates were identified as meropenem-nonsusceptible (aztreonam MIC_50_ and MIC_90_ values both >128 mg/L) [24]. In that study, avibactam effectively restored aztreonam efficacy and reduced MIC_50_ and MIC_90_ values to 0.25 mg/L and 1 mg/L, respectively. A separate global study of aztreonam–avibactam antimicrobial activity involved 1498 meropenem-nonsusceptible Enterobacteriaceae clinical isolates collected in 40 countries in 2017 [19]. MIC_50_ and MIC_90_ values for the aztreonam monotherapy were both >128 mg/L. Aztreonam–avibactam results included an MIC_50_ of 0.25 mg/L and MIC_90_ of 1 mg/L (99.2% susceptibility, 1486/1498). In a study involving 177 carbapenemase-producing Enterobacteriaceae isolates collected in Singapore and the US [25], MIC_50_ and MIC_90_ values for aztreonam alone against different carbapenemase classes were 128–512 mg/L and >512 mg/L, respectively. For the combination of aztreonam and avibactam they ranged from 0.12 to 0.25 mg/L and from 0.5 to 1 mg/L, also respectively. We found that avibactam significantly restored aztreonam activity (*p* < 0.0001) (Table 1 and Figure 2), with high levels of CnsEC (94.1%) and CnsKP (95.3%) susceptibility (Figure 3b,d).

Based on evidence showing β-lactamases (both *bla*_AmpC_ and carbapenemases) as contributing to carbapenem resistance [2], β-lactamase inhibitors such as avibactam and relebactam have been examined as candidates for treating carbapenem-resistant Gram-negative bacilli [5]. Avibactam and relebactam belong to a class of bi-cyclic diazabicyclooctane β-lactamase inhibitors that only act against serine β-lactamases [22,26]. Specifically, avibactam is active against class A, C, and D β-lactamases [27], and relebactam mostly inhibits class A and C and a small number of class D β-lactamases [28]. Aztreonam, which is active against metallo-β-lactamase- (MBL-) producing bacteria, is subject to hydrolyzation by class A or D β-lactamases [23]. The combination of aztreonam with avibactam (a class A or D β-lactamase inhibitor) expresses antimicrobial activity against bacteria that carry MBL with class A or D β-lactamases [29].

In a previous study, 177 carbapenemase-producing Gram-negative bacilli isolates (116 class A, 47 class B, and 14 class D) were examined to determine ceftazidime–avibactam and aztreonam–avibactam susceptibilities [25]. Both combinations were found to be effective against all class A (108 *bla*_KPC_, 5 *bla*_IMI_ and 3 *bla*_SME_) and class D carbapenemase isolates, with susceptibilities ranging from 93% to 100%. In comparison, low susceptibility values were noted for all 47 class B carbapenemase isolates (32 *bla*_NDM_, 11 *bla*_IMP_, and 4 *bla*_VIM_) treated with ceftazidime–avibactam (0–9%). High susceptibility values were observed following aztreonam–avibactam treatment (94–100%). In summary, we found that the ceftazidime–avibactam combination was generally ineffective against class B carbapenemase-producing isolates, while the aztreonam–avibactam combination exhibited robust efficacy in all carbapenemase-producing isolate classes (Table 1; Figure 4). Ceftazidime–avibactam activity was restored by the addition of EDTA, further evidence of the MBL effect (Figure 5).

Several research teams have described the efficacy of the ceftazidime–avibactam combination in vivo [30,31,32], but little is known about the combination of aztreonam with avibactam. In one study involving mice infected with carbapenemase-producing *K. pneumoniae*, 100% of those treated with ceftazidime–avibactam survived, and 70% treated with a placebo died within 4 days [30]. In a retrospective clinical study, the 30-day mortality rate for 104 patients infected with *bla*_KPC_-carrying *K. pneumoniae* decreased significantly following treatment with ceftazidime–avibactam (*p* = 0.005, 36.5% vs. 55.8% for other therapies) [31]. For our study we infected a *C. elegans* model in vivo with a randomly selected CRE-1462 *bla*_KPC_-containing *K. pneumoniae* clinical isolate and measured the effects of treatment with either ceftazidime–avibactam or aztreonam–avibactam. Significant right-shifts in survival curves were observed in both treatment groups (both *p* < 0.0001) (Figure 5), with extended median survival times of 2–4 days (Table 2). In addition to suggesting the in vivo efficacy of ceftazidime–avibactam, our data also indicate in vivo aztreonam–avibactam efficacy against a *bla*_KPC_-producing *K. pneumoniae* clinical isolate.

## 5. Conclusions

Our data indicate therapeutic effectiveness for ceftazidime–avibactam and aztreonam–avibactam combinations against carbapenem-nonsusceptible Enterobacteriaceae, with respective susceptibilities of 87.0% (574/660) and 95.0% (627/660). The aztreonam–avibactam combination in particular seems to exert a powerful antibacterial effect against metallo-carbapenemase-producing Enterobacteriaceae, but further clinical research is required for confirmation.

## Figures and Tables

**Table 1:** Minimum inhibitory concentration (MIC) values for ceftazidime–avibactam and aztreonam–avibactam combinations.

Bacterium	Group	MIC	Antimicrobial Agent and *p* Value ^a^							
			CAZ	CAZ-AVI	Mean log_2_ MIC Change (95% CI)	*p* ^b^	AZT	AZT-AVI	Mean log_2_ MIC Change (95% CI)	*p* ^b^
*Klebsiella pneumoniae*	Total (*n* = 472)	Range	1 ~ >64	<0.06 ~ >64	−5.3 (−5.5, −5.2)	<0.0001	0.125 ~ >32	<0.06 ~ >32	−6.0 (−6.1, −5.8)	<0.0001
		MIC_50_	>64	2			>32	0.5		
		MIC_90_	>64	8			>32	2		
		% susceptible	0.4% (2/472)	91.5% (432/472)			7.4% (35/472)	95.3% (450/472)		
	Class A carbapenemase (*n* = 121)	Range	8 ~ >64	0.25 ~ >64	−5.2 (−5.4, −5.0)	<0.0001	16 ~ >32	0.125 ~ 8	−6.3 (−6.5, −6.2)	<0.0001
		MIC_50_	>64	2			>32	1		
		MIC_90_	>64	8			>32	2		
		% susceptible	0% (0/121)	95.0% (115/121)			0% (0/121)	99.2% (120/121)		
	Class B carbapenemase (*n* = 14)	Range	32 ~ >64	1 ~ >64	−0.6 (−1.4, 0.3)	0.1788	0.125 ~ >32	<0.06 ~ >32	−6.0 (−8.2, −3.8)	<0.0001
		MIC_50_	>64	>64			>32	0.125		
		MIC_90_	>64	>64			>32	0.5		
		% susceptible	0% (0/14)	7.1% (1/14)			21.4% (3/14)	92.9% (13/14)		
	Class D carbapenemase (*n* = 10)	Range	8 ~ 64	0.25 ~ 2	−4.8 (−5.7, −3.9)	<0.0001	1 ~ >32	0.125 ~ 2	−6.0 (−7.2, −4.8)	<0.0001
		MIC_50_	16	1			32	0.25		
		MIC_90_	64	2			>32	2		
		% susceptible	0% (0/10)	100% (10/10)			10% (1/10)	100% (10/10)		
	Non-carbapenemase producer (*n* = 329)	Range	1 ~ >64	<0.06 ~ >64	−5.6 (−5.8, −5.4)	<0.0001	0.25 ~ >32	<0.06 ~ >32	−5.8 (−6.0, −5.6)	<0.0001
		MIC_50_	>64	1			>32	0.5		
		MIC_90_	>64	8			>32	4		
		% susceptible	0.6% (2/329)	93.6% (308/329)			9.4% (31/329)	93.9% (309/329)		
*Escherichia coli*	Total (*n* = 188)	Range	0.125 ~ >64	<0.06 ~ >64	−6.6 (−7.0, −6.3)	<0.0001	<0.06 ~ >32	<0.06 ~ 32	−6.3 (−6.6, −6.0)	<0.0001
		MIC_50_	>64	0.5			>32	0.5		
		MIC_90_	>64	4			>32	4		
		% susceptible	2.1% (4/188)	93.6% (176/188)			3.2% (6/188)	94.1% (177/188)		
	Class A carbapenemase (*n* = 3)	Range	8 ~ 32	0.125 ~ 2	−5.3 (−8.2, −2.5)	0.0153	32 ~ >32	<0.06 ~ 0.125	−9.0 (−9.2, −8.9)	<0.0001
		MIC_50_	8	0.125			32	<0.06		
		MIC_90_	32	2			>32	0.125		
		% susceptible	0% (0/3)	100% (3/3)			0% (0/3)	100% (3/3)		
	Class B carbapenemase (*n* = 9)	Range	32 ~ >64	<0.06 ~ >64	−1.1 (−3.4, 1.2)	0.2953	2 ~ >32	<0.06 ~ 2	−8.1 (−9.6, −6.6)	<0.0001
		MIC_50_	>64	>64			32	<0.125		
		MIC_90_	>64	>64			>32	2		
		% susceptible	0% (0/9)	11.1% (1/9)			11.1% (1/9)	100% (9/9)		
	Class D carbapenemase (*n* = 2)	Range	>64	0.25 ~ 4	−	−	>32	0.125 ~ 4	−	−
		MIC_50_	>64	0.25			>32	0.125		
		MIC_90_	>64	4			>32	4		
		% susceptible	0% (0/2)	100% (2/2)			0% (0/2)	100% (2/2)		
	Non-carbapenemase producer (*n* = 174)	Range	0.125 ~ >64	<0.06 ~ 16	−6.9 (−7.2, −6.7)	<0.0001	<0.06 ~ >32	<0.06 ~ 32	−6.2 (−6.5, 5.9)	<0.0001
		MIC_50_	>64	0.5			>32	0.5		
		MIC_90_	>64	2			>32	4		
		% susceptible	2.3% (4/174)	97.7% (170/174)			2.9% (5/174)	93.7% (163/174)		

Note: Clinical and Laboratory Standards Institute (CLSI) interpretive criteria for single-agent aztreonam was used to interpret the susceptibility of aztreonam–avibactam combination. ^a^ Abbreviations: CAZ, ceftazidime; CAZ-AVI, ceftazidime–avibactam; AZT, aztreonam; AZT-AVI, aztreonam–avibactam. ^b^ *p* values were analyzed via the MIC data.

**Table 2:** In vivo *C. elegans* statistical data.

Treatment	Median Survival Time (Days)	*p* Value	Hazard Ratio		
			Ratio	Lower 95%	Upper 95%
untreated control	2	−	−	−	−
ceftazidime	2	−	1	−	−
ceftazidime–avibactam	4	<0.0001	0.472	0.295	0.756
aztreonam	2	−	1	−	−
aztreonam–avibactam	4	<0.0001	0.420	0.260	0.679

Note: All experiments were performed in triplicate.
